# Gut Microbiota in Patients with Different Metabolic Statuses: Moscow Study

**DOI:** 10.3390/microorganisms6040098

**Published:** 2018-09-25

**Authors:** Daria A. Kashtanova, Olga N. Tkacheva, Ekaterina N. Doudinskaya, Irina D. Strazhesko, Yulia V. Kotovskaya, Anna S. Popenko, Alexander V. Tyakht, Dmitry G. Alexeev

**Affiliations:** 1 Russian Clinical Research Center for Gerontology, Pirogov Russian National Research Medical University of the Ministry of Healthcare of the Russian Federation, bld. 16, 1st Leonova Street, Moscow 129226, Russia; tkacheva@rambler.ru (O.N.T.); katharina.gin@gmail.com (E.N.D.); kotovskaya@bk.ru (Y.V.K.); 2 Medical Research and Education Center, Lomonosov Moscow State University, 1 Leninskie Gory, Moscow 119991, Russia; Istrazhesko@gmail.com; 3 Atlas Biomed Group, Tintagel House, 92 Albert Embankment, Lambeth, London SE1 7TP, UK; kirika77@gmail.com (A.S.P.); a.tyakht@gmail.com (A.V.T.); Dmitry.g.alexeev@gmail.com (D.G.A.)

**Keywords:** gut microbiota, metabolic status, glucose metabolism, cardiovascular risk factors, diet

## Abstract

The aim of this paper was to study gut microbiota composition in patients with different metabolic statuses. Methods: 92 participants aged 25–76 years (26 of whom were men), with confirmed absence of cardiovascular and other chronic diseases (but with the possible presence of cardiovascular risk factors) were included. Carotid ultrasound examinations, 16*S* rRNA sequencing of stool samples and diet assessments were performed. Statistical analysis was performed using R programming language, 3.1.0. Results: Enterotyping yielded two clusters differentiated by alpha-diversity. Intima-media thickness was higher in the cluster with lower diversity (adj. *p* < 0.001). Obesity was associated with higher *Serratia* (adj. *p* = 0.003) and *Prevotella* (adj. *p* < 0.0003) in relative abundance. Abdominal obesity was associated with higher abundance of *Serratia* (adj. *p* = 0.004) and *Prevotella* (adj. *p* = 0.0008) and lower levels of *Oscillospira* (adj. *p* = 0.0005). Glucose metabolism disturbances were associated with higher *Blautia* (adj. *p* = 0.0007) and *Serratia* (adj. *p* = 0.003) prevalence. Arterial hypertension was associated with high *Blautia* levels (adj. *p* = 0.002). The *Blautia* genus strongly correlated with low resistant starch consumption (adj. *p* = 0.007). A combination of high-fat diet and elevated *Blautia* levels was very common for diabetes mellitus type 2 patients (adj. *p* = 0.0001). Conclusion: The results show that there is a relationship between metabolic changes and higher representation of opportunistic pathogens and low diversity of gut microbiota even in apparently healthy participants.

## 1. Introduction

The leading cause of death in the world is still cardiovascular disease (CVD) and associated metabolic conditions, despite all healthcare measures that have been undertaken. Modern medicine requires a preventive, personalized approach and early preclinical diagnosis. By taking early action for the prevention of cardiovascular risk factors, CVD can, in time, be prevented or delayed.

Scientific progress in molecular genetic analysis techniques allows the detailed examination of gut microbiota community structure and functions. It turns out that gut microbiota is strongly involved in many aspects of human body functions, having a significant effect on immunity, metabolism and even the nervous system and higher nervous activity [1]. Some previous studies had shown a relationship between gut microbiota and obesity [2]. In the present study, associations between the composition of gut microbiota, metabolic markers and diet were studied in a carefully examined cohort without clinical CVD or other chronic diseases.

**Aim:** To assess the association between intestinal microbiota composition and metabolic markers in apparently healthy individuals.

## 2. Materials and Methods

The study included Caucasian participants (Moscow and Moscow Region) aged 25 to 76 years, after the preventive outpatient screening.

### 2.1. The Inclusion Criteria

The study included men and women aged over 25 years, who had not been treated with any drugs (for at least 6 months) and who had no clinical cardiovascular or other chronic diseases. All patients signed informed consent forms.

### 2.2. The Exclusion Criteria

Reasons for exclusion included unsigned consent forms or refusal to participate in the study; coronary artery disease, including myocardial angina, infarction, cerebrovascular disease, intermittent claudication, stroke, etc., as well as any other clinical cardiovascular diseases (arrhythmia, valvular heart disease, etc.); treatment with any drug or nutraceuticals (including antibiotics and probiotics during the last 3 months); diabetes mellitus type 1 or other specific types of diabetes, history of diabetes mellitus type 2 (DM-2; excluding new-onset DM-2 diagnosed with oral glucose tolerance test before the study); anemia; cancer history; chronic liver and kidney diseases; pregnancy or lactation; infectious diseases (during the previous 3 months); abdominal surgery; acute or chronic diseases of gastrointestinal tract; diagnosed enzymatic deficiency and food allergies; dental or oral cavity diseases; organ transplantation history. Patients were excluded from the study at any time if any exclusion criteria were met.

### 2.3. Ethical Aspects

Informed consent was given by all participants. Data privacy was ensured by using anonymized identifiers. Protocol of the study was approved by the local ethics committee, meeting #8, 29 November 2011.

### 2.4. Patients’ Preassessment

Participants underwent a thorough assessment before inclusion. Preassessment included physician and other specialists consultations (cardiologist, stomatologist), anthropometric measurement, complete blood count, blood biochemistry (glucose, creatinine, urea, uric acid, potassium, sodium, bilirubin, transaminases, total cholesterol (TC), high-density lipoproteins (HDL) and low-density lipoproteins (LDL), triglycerides (TG) and thyroid-stimulating hormone, urinalysis, electrocardiogram (Schiller Cardiovit AT-10, Schiller, Baar, Switzerland), and Treadmill Test (Intertrack, Schiller, Baar, Switzerland)). Those with any deviations in examinations were not included in the study. Metabolic risk markers were as follows:

- Glucose metabolism impairment: fasting hyperglycemia (fasting venous plasma glucose ≥6.1 and <7.0 mmol/L) or impaired glucose tolerance (IGT) (fasting venous plasma glucose <7 mmol/L, ≥7.8 and <11.1 mg/dL 2 h after oral glucose tolerance test (OGTT)) or new onset DM-2 (fasting glucose ≥7 mmol/L or ≥11.1 mmol/L 2 h after OGTT);
- Lipid metabolism impairment: TC >5.0 mmol/L and/or LDL >3.0 mmol/L and/or HDL <1.0 mmol/L for men and <1.2 mmol/L for women and/or triglycerides >1.7 mmol/L;
- Body Mass Index (BMI) ≥ 30 kg/m^2^ (obesity) and/or waist circumference ≥94 cm for men and ≥80 cm for women (abdominal obesity);
- Arterial hypertension (AH) (systolic/diastolic blood pressure (BP) level of 140/90–159/99 mmHg). Patients with BP 160/100 mmHg and higher and those who required antihypertensive treatment were not included;
- Smoking (regardless of the numbers of cigarettes and years of smoking).

### 2.5. Basic Methods

#### 2.5.1. Gut Microbiota Composition

Stool samples were frozen and stored at −20 °C and then thawed. To assess microbiota composition, sequencing of the 16*S* rRNA gene variable V3–V4 regions was performed (subsequent to total DNA isolation and library preparation) by using the MiSeq Reagent Kit v2 and MiSeq sequencer (Illumina, San Diego, California, CA, USA) according to the company’s recommendations.

#### 2.5.2. Library Preparation

Libraries were prepared with ‘16*S* Metagenomic Sequencing Library Preparation: Preparing 16*S* Ribosomal RNA Gene Amplicons for the Illumina MiSeq System’ protocol (15044223 Rev. B) using the Nextera XT Index Kit (Illumina) with a dual indexing strategy.

#### 2.5.3. Blood Vessels Assessment

Vessels assessment was carried out using Q-LAB (Philips, Eindhoven, The Netherlands) during carotid artery duplex scan in B-mode with ECG recording. To assess common carotid artery (CCA) intima-media thickness (IMT), European Society of Hypertension and the European Society of Cardiology (2003) standards were used. IMT <0.9 mm was considered to be a normal thickness; 0.9–1.3 mm indicated increased thickness. Atherosclerosis was defined as IMT >1.3 mm or a local increase in IMT of 0.5 mm or a 50% increase in nearby IMT. Plaque was considered as a local IMT thickening >1.0 mm, which caused lumen stenosis but did not affect its internal anatomy [3].

### 2.6. Bioinformatic Processing

Quality filtering reads and taxonomic classification were performed by using QIIME Software v. 1.7 [4]. Taxonomic composition of the samples was assessed using the Greengenes v. 13.5 database (http://greengenes. secondgenome.com/downloads/database/13_5) and RDP Classifier. Operational taxonomic units (OTUs) levels were determined in analysis. Bioinformatic processing was performed using R programming language (version 3.1.0, R Core Team, Auckland, New Zealand). Statistical evaluation was performed using generalized linear models and Mann–Whitney tests (false discovery rate (FDR)), sex and age adjusted) [5].

## 3. Results

**Figure 1:**
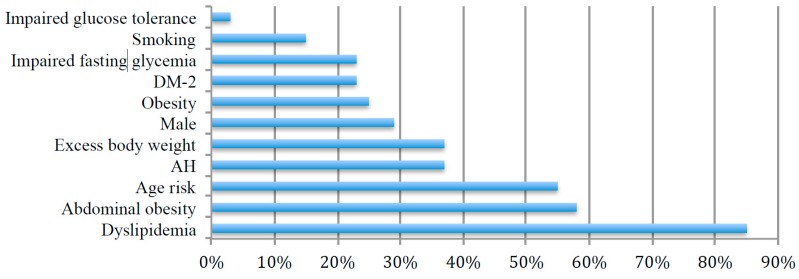
Prevalence of cardiovascular risk factors in participants. DM-2—diabetes mellitus type 2, AH—arterial hypertension.

Ninety-two participants aged 25–76 years (of which 26 were men) were included. Their average age was 52 ± 13 years (53 ± 13 years for women and 51 ± 13 years for men). The most common metabolic disorder was dyslipidemia, which was diagnosed in 85% of participants; abdominal obesity was diagnosed in 58%; first grade hypertension in 37%; new onset DM-2 in 23%. The prevalence of risk factors is displayed in Figure 1.

### 3.1. Gut Microbiota Assessment

An average of 102,582 ± 46,284 reads per sample were detected. The short- and low-quality reads (median QV < 20) were removed; a total of 102,581 ± 39,210 high quality reads (87 ± 2% of the initial amount) were analyzed. Raw sequencing data have been set out in a previous paper [6]. Of those, 87.40 ± 7.4% were classified taxonomically. Among them, 97.41 ± 0.9% were classified to the genus level. Quality criteria were met for the sequencing results. Bacteroidetes (12.7 ± 9.86%) and Firmicutes (57.09 ± 13.6%) were the dominant phyla in all study samples. *Blautia*, *Bacteroides*, *Prevotella*, *Faecalibacterium* and *Clostridium* were the most represented genera (more than 50% of microbial abundance). *Blautia* was the most dominant genus.

### 3.2. Cluster Analysis

**Figure 2:**
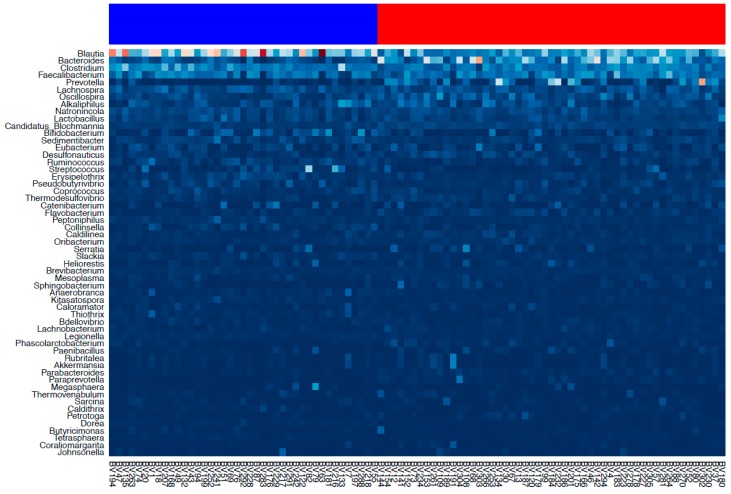
Heatmap of the genus-level taxonomic composition (only the major genera forming 85% of the total abundance are displayed). First cluster samples are marked in red color, second cluster in blue.

Cluster analysis was carried out according to the enterotyping algorithm [7]. As a result, the samples were divided into two clusters: 52 samples in the first cluster, 40 in the second (Figure 2).

These clusters were not clearly separated: the value of the average silhouette was 0.22 while the commonly used threshold value is 0.8 [8]. Clusters were not distinguished by age or sex of the subjects, but the proportions of subjects with DM-2 (adj. *p* = 0.015) and with intima-media thickening (adj. *p* = 0.04) were significantly higher in the second cluster. The occurrence of DM-2 in the second cluster was 32.5% compared to 13.5% in the first cluster. Intima-media thickening (>0.9 mm) was determined in 30% of patients in the second cluster compared to 11.5% in the first cluster (adj. *p* = 0.04). There was a significant difference in the distribution of nine bacterial genera (Table 1) between the clusters.

**Figure 3:**
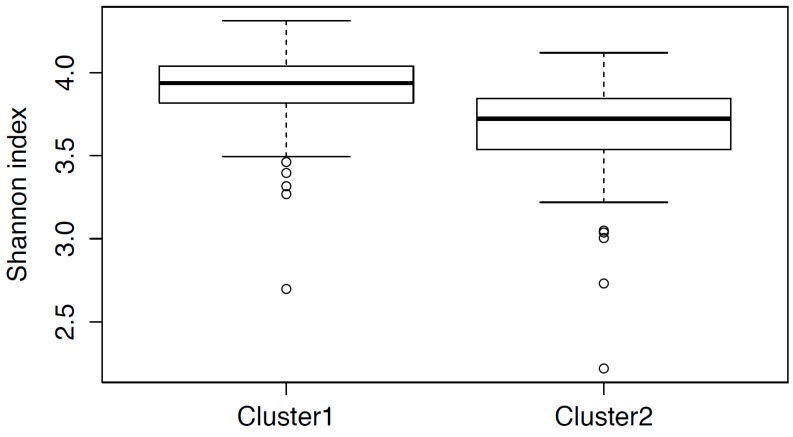
Distribution of alpha-diversity across the microbiota clusters.

Alpha-diversity strongly differed in these clusters also in their distribution of (Figure 3, *p* < 0.001). In the first cluster, the diversity was higher; this characterizes the gut microbiota composition of these donors as potentially more stable and resistant to external influences.

### 3.3. Cardiovascular Risk Factors and Gut Microbiota Composition

When studying metabolic status and its association with the microbiota composition, it was found that the amount of cardiovascular risk factors correlated with the predominance of the opportunistic [9] *Serratia* genus, the number of which was significantly (adj. *p* < 0.001) higher in patients with a greater number of risk factors.

#### 3.3.1. Obesity and Gut Microbiota

The average BMI was 27.31 ± 5.4 kg/m^2^. Excess body weight was diagnosed in 34 (37%) participants, obesity in 23 (25%). Abdominal obesity was detected in 53 (58%) participants. The average waist size was 89 ± 14.95 cm.

Abdominal obesity, overweight and obesity were associated with an increased abundance of *Serratia* and *Prevotella* genera in gut microbiota. An inverse relationship between abdominal obesity and the presence of an amylolytic *Oscillospira* taxon was observed (Table 2).

#### 3.3.2. Lipids and Gut Microbiota

Dyslipidemia was the most common metabolic disorder in the studied group and was noted in 78 (85%) participants. There was no difference in the gut microbiota composition in patients with and without dyslipidemia regardless of the criterion for its diagnosis: increased total cholesterol, LDL cholesterol, TG, reduced HDL cholesterol, or a combination of these criteria (generalized linear modeling (GLM)).

#### 3.3.3. Glucose Metabolism and Gut Microbiota

The average fasting glucose level was 5.74 ± 1.53 mmol/L. Glucose metabolism disorders were noted in 24 subjects (26%): impaired fasting glycemia (IFG) in 21 (23%); impaired glucose tolerance (IGT) in three (3%). DM-2, according to the data of OGTT, was detected in 21 (23%) people. DM-2 was associated with higher prevalence of the *Blautia* genus (adj. *p* = 0.007), and there was a significant increase in the number of these bacteria when the carbohydrate metabolism was disrupted (normal carbohydrate metabolism prediabetes (IFG + IGT) DM-2 groups, adj. *p* = 0.0007). Regression analysis showed that in these clinical series there was a significant increase in the abundance of the genus *Serratia* (adj. *p* = 0.003).

#### 3.3.4. First Grade Hypertension and Gut Microbiota

Arterial hypertension of the first degree was detected in 34 (37%) participants. GLM showed that individuals with elevated systolic blood pressure (SBP) had a higher abundance of *Prevotella* than those with normal SBP (adj. *p* < 0.001). Taking into account the association between gut microbiota and DM-2, the group without glucose metabolism impairments was assessed. In this analysis it was found that among persons without DM-2, SBP was much higher in patients whose intestinal microbiota contained more bacteria of the *Blautia* genus (adj. *p* = 0.002).

#### 3.3.5. Smoking and Gut Microbiota

In the study, there were no significant differences in microbiota composition between smokers and non-smokers. Perhaps this is due to the disproportionately small size of this subgroup: only 14 (15%) of the subjects were smokers.

#### 3.3.6. Diet and Gut Microbiota

Among bacteria associated with clinical parameters, *Blautia* (associations with first grade arterial hypertension, DM-2) abundance was significantly associated with resistant starch consumption (adj. *p* = 0.007). Notably, high consumption of cholesterol, as well as of alcohol, was strongly associated with a lower abundance of *Bifidobacterium* (adj. *p* = 0.008 and 0.006, respectively). 

To analyze dietary habits, the subjects were divided into two clusters using the k-medoid algorithm with Bray–Curtis distance matrix, calculated from the percentage of proteins, fats and carbohydrates in the donors’ diets according to food frequency questionnaires. The first cluster contained 64 samples, the second, 22.

These clusters were significantly different in relation to the percentage of proteins, fats and carbohydrates in the donors’ diets (*p* < 0.001 for each nutrient). The first dietary cluster was characterized by a greater proportion of carbohydrates in the diet, the second by a greater proportion of fats and proteins. In the second dietary cluster, the proportion of samples from donors with newly diagnosed DM-2 was two times greater (15.6% in the first cluster and 32% in the second cluster).

The dietary clusters were not differentiated by age, gender or BMI of their subjects (*p* = 0.5, 0.25, 0.4, respectively). In the second dietary cluster, a difference between samples from healthy donors and donors with DM-2 was found in the *Prevotella* genus abundance (higher in the DM-2 group, adj. *p* < 0.001). However, the trend was not obvious, possibly due to a wide spread in the nutrient composition of the diet (for example, donors with impaired glucose metabolism in the second diet cluster consumed more calcium (*p* = 0.03)). Therefore, a similar method of searching for optimal dietary clusters was used, but the number of clusters was determined by the Calinski–Harabasz index, in order to identify samples with more similar diets. Ten clusters were identified.

Donors in one of the clusters had a very similar diet, consuming a lot of fats, although half of them had normal glucose metabolism and the other half were newly diagnosed with DM-2. There were no differences in age, gender or quantity of consumed calories between these two groups (*p* = 0.8, 0.5 and 0.3 respectively), nor in other nutritional features.

GML analysis showed that *Blautia* genus abundance (adj. *p* = 0.0001) was higher in the samples from donors with DM-2, which is consistent with previous analyses [6].

## 4. Discussion

The central observation of our study was that a lot of associations with clinical parameters were found for gram-negative opportunistic pathogens. These bacteria are normally present in the microbiota but an imbalance, and their increased presence, is associated with increased permeability of the intestinal epithelium, the release of lipopolysaccharide, endotoxemia and low-grade inflammation. *Serratia* and *Prevotella* genera were more abundant among participants with obesity, high blood pressure and impaired glucose metabolism. Their metabolites initiate inflammation by activating the production of interleukins, the nuclear factor NF-κB, and other inflammatory markers by stimulating proteinases of activated type 2 receptors [9,10]. Another interesting genus was *Blautia*, which was one of the most common, and was associated with increased blood pressure and glucose metabolism impairment. According to the study by Tuovinen et al. [11] *Blautia coccoides* activate the secretion of tumor necrosis factor α, cytokines and interleukin-8 even more than lipopolysaccharide. The keystone-pathogen hypothesis identifies the possible metabolic roles of low-abundance microbial pathogens in the gut [12]. At the same time, the combination of high *Blautia* abundance with a high-fat diet was very common in DM-2 patients. However, larger prospective studies should be conducted to confirm this hypothesis. It is known that diet affects gut microbiota composition, which influences the growth of beneficial/opportunistic bacteria and can become an instrument to individualize a diet considering the initial bacteria composition [13].

*Oscillospira* genus was less present in participants with obesity. This confirms an earlier study conducted in monozygotic twins, which showed a strong inverse relationship of *Oscillospira* bacteria with obesity [14]. The authors emphasize that bacteria of this genus break down complex carbohydrates, i.e., their large number indicates a relatively “healthy” diet. Amylolytic bacteria can produce short-chain fatty acids that have an anti-inflammatory effect, inhibit nuclear factor kB activity, reduce the synthesis of pro-inflammatory interleukins, participate in motor regulation and perform a detoxification function.

As is known, low-grade inflammation is a common link for various disorders, including metabolic syndrome and atherosclerosis. Thus, it is likely that gut microbiota is associated with metabolic disorders throughout the immune system by suppressing or stimulating inflammation. 

Our results show that there is a relationship between metabolic changes and a higher abundance of opportunistic pathogens in gut microbiota and its low diversity even in apparently healthy participants. We propose that the “gut–heart axis” may be involved in the very early stages of cardiovascular diseases.

## Figures and Tables

**Table 1:** The genera differentially abundant in the two microbiota clusters.

	Genus	Mann–Whitney U Test, FDR Adjusted *p*-Value
**1**	Prevotella *	0.0001
**2**	Oscillospira *	0.005
**3**	*Ruminococcus*	0.008
**4**	Flavobacterium *	0.005
**5**	*Peptoniphilus*	0.005
**6**	Sphingobacterium *	0.0003
**7**	*Thiothrix*	0.005
**8**	*Legionella*	0.005
**9**	Parabacteroides *	0.002

* Higher bacteria abundance in the first cluster. Higher bacteria abundance in the second cluster.

**Table 2:** Links of gut microbial genera levels with body mass index (BMI) and abdominal obesity according to generalized linear models modeling results.

	Genus	Estimate	Standart Error	z Value	FDR
Obesity	*Serratia*	0.64	0.17	3.85	0.003
Obesity	*Prevotella*	0.56	0.12	4.54	0.0003
Abdominal obesity	*Prevotella*	0.49	0.12	4.19	0.0008
Abdominal obesity	*Oscillospira*	−0.51	0.11	−4.44	0.0005
Abdominal obesity	*Serratia*	1.20	0.33	3.67	0.004
